# Mandibular Third Molar Impaction and Bone Change Distal to the Second Molar: A Panoramic Radiographic Study

**DOI:** 10.3390/jcm13030906

**Published:** 2024-02-04

**Authors:** Hassan Assiri Ahmed, Jose López-López, Sonia Egido-Moreno, Xavier Roselló Llabrés, Mohammed Hameed, Albert Estrugo-Devesa

**Affiliations:** 1 Department of Odontostomatology, Faculty of Medicine and Health Sciences, School of Dentistry, University Campus of Bellvitge, University of Barcelona, Barcelona Dental Hospital [HOUB], 08970 Barcelona, Spain or dinho.1010@hotmail.com (H.A.A.); soniaegido@ub.edu (S.E.-M.); xavierroselloll@ub.edu (X.R.L.); albertestrugo@ub.edu (A.E.-D.); 2 Department of Diagnostic Science and Oral Biology, Faculty of Dentistry, King Khalid University, Abha 61421, Saudi Arabia; mhmead@kku.edu.sa

**Keywords:** impacted mandibular third molar, bone loss, mandibular second molar, panoramic radiography

## Abstract

**Background:** The mandibular third molar is the most frequently impacted tooth. An impacted mandibular third molar (IMTM) can have negative consequences on the adjacent mandibular second molar (MSM), such as bone loss. An IMTM can be identified using orthopantomography (OPG). Our objective is to compare changes in bone level distal to the mandibular second molar (MSM) in patients with an extracted IMTM versus non-extracted IMTM using OPG. **Methods:** In this retrospective case–control study, 160 orthopantomograms (OPGs) of 80 patients who attended Dental Hospital of the University of Barcelona (HOUB) were randomly selected. Participants were stratified into a study group and control group. **Results:** Males and females experienced bone gain in the study group and bone loss in the control group. However, the difference in bone-level change was not statistically significant regarding gender in the study group. Within the study group, the age group of 29–39 years demonstrated significant (*p*-value = 0.042) bone gain after extraction compared to other age groups. However, the control group demonstrated bone loss in all age groups in which the difference is not statistically significant (*p*-value 0.794). **Conclusions:** Bone improvements distal to the MSM were observed after the extraction of an IMTM compared to when an IMTM was not extracted.

## 1. Introduction

The term “erumpere”, which means “to erupt”, refers to the movement of a developing tooth from its non-functional position to its corresponding functional and anatomical position in the dental arch [1]. However, for several reasons, some teeth fail to undergo this physiological process, leading to partial or complete tooth impaction [1]. An unerupted tooth is defined as one that remains embedded within the jawbone, is covered by gingival tissue, and may be partially or entirely covered by bone. Nevertheless, such a tooth is expected to erupt and come into occlusion based on clinical and radiographic findings [2]. A partially erupted tooth is defined as one that does not fully erupt into its normal position but can still be seen in the oral cavity. The term “tooth impaction” signifies that a tooth is obstructed from fully erupting into the oral cavity due to the blockage of the eruption path or a lack of space [3]. The third molars, followed by the maxillary canines, are the most frequently impacted teeth that are routinely encountered in dental practice [3]. The frequency of mandibular third molar impaction is influenced by factors such as facial skeleton, gender, age, and ethnic group [4]. Breik and Grupor [4] reported a higher incidence of mandibular third molar impaction in individuals with a dolichofacial pattern (vertical growth; facial axis angle, <87) and in females (43%) compared to males (45%). On the contrary, Padhye et al. [5] observed a higher prevalence of third molar impaction in males (51.77%) than in females (48.33%). The study by KalaiSelvan et al. [6] reported a prevalence of 45.8% in the Tamil population, while Prajapati et al. [7] reported a higher prevalence of impacted mandibular third molars (IMTMs) among females aged 21–30. A study by Selene Barone et al. [8] reported a statistically significant correlation between the Gonial angle and the position of the mandibular third molar. They observed that with a progressive decrease in the Gonial angle, a higher incidence of IMTMs was observed (*p* < 0.05). The worldwide prevalence of impacted third molars is 24% [9].

Systemic local reasons and craniofacial development contribute to tooth impaction. Systemic conditions, such as nutritional deficiencies, vitamin D insufficiency, anemia, rickets, Down’s syndrome, genetic factors, endocrine disturbances, and various syndromes and infectious diseases, play a significant role in tooth impaction [9]. Local factors and craniofacial development that can hinder eruption and lead to impaction include a premature loss of deciduous teeth, traumatic conditions, ankylosed teeth, inflammatory and pathological conditions, Gonial angle and an insufficient space in the dental arch [1,8,9].

Different imaging modalities, including conventional radiography and advanced three-dimensional imaging, are used in dental practice. The initial screening of oral-cavity-related abnormalities is performed using periapical and panoramic radiography. Intraoral periapical radiography is used whenever it is possible to position a radiographic sensor inside the oral cavity. This technique provides a sharp and detailed image of the impacted tooth and its relation to the inferior alveolar canal [10]. The tube-shift technique can be used to determine the relationship between a tooth and the inferior mandibular nerve [10]. However, due to the difficulties associated with positioning the device, panoramic radiography is more convenient to use [11]. An orthopantomogram (OPG) provides the advantage of being able to view the structures of the mandible, maxilla, and facial bone in a single broad image. It is beneficial for identifying different pathological conditions, assessing carious and fractured teeth, detecting dental anomalies, and determining the presence of impacted teeth and pathologies [2]. However, there are several drawbacks linked to the panoramic radiographic technique, including overlapping, magnification, blurred images, metal artifacts, and errors related to patient positioning and image acquisition [11]. Therefore, the need for more accurate three-dimensional diagnosis has led to the introduction of cone-beam computed tomography (CBCT). CBCT is considered superior to conventional panoramic radiography because it provides a three-dimensional view of structures [12]. However, the routine use of CBCT in dental practice is controversial and limited to patients’ needs since it produces higher radiation doses than conventional dental radiography [12]. The radiation doses resulting from a full field-of-view dental CBCT scan are reported to be 4–42 times higher than the doses from an OPG [13]. Thus, it is necessary to implement the ALADA (as low as diagnostically acceptable) concept, which has replaced the previous ALARA (as low as reasonably acceptable) concept, to control the use of CBCT [14]. In this study, IMTM was the primary predictor variable and bone loss in the adjacent MSM was the outcome variable. The null hypothesis was that there is no difference in bone in the adjacent MSM, regardless of the extraction of the IMTM.

Hence, this study aimed to compare changes in the bone level between patients who underwent the extraction of an IMTM and those who did not. The findings were also analyzed in relation to the gender and age of the patients. Additionally, the pattern of impaction in the study sample was examined.

## 2. Materials and Methods

### 2.1. Study Description

This retrospective case–control study utilized archived panoramic radiographic images of patients who visited the Dental Hospital of the University of Barcelona (HOUB) from June 2018 to August 2022. A total of 1000 OPGs were randomly selected. Among them, 400 were deemed error-free and suitable for analysis. Based on the study characteristics, 160 OPGs were selected to perform the measurements; these OPGs were from 40 patients representing the study group and 40 patients representing the control group. Ethical approval for this study was obtained from the ethics committee of HOUB (protocol number: 2022-032-1).

### 2.2. Study Description

The patients were selected using a simple random sampling technique, and the sample size was calculated based on Cohen’s concept, as described in a study conducted by Faria et al. [15]. The minimum determined sample size for each group was 24; however, 80 patients (40 in each group) were included in this study to enhance validity. The patients were divided into two groups based on whether they underwent an extraction (study group) or non-extraction (control group) of the IMTM. The patients in the study group were individuals who came to the clinic to have their impacted lower third molar extracted. Two X-rays were taken for evaluation, one before the extraction and the other at least 3–6 months later, given that the time necessary for bone healing and remodeling is three months. This period is considered the cut-off period for periodontal healing [16]. The measurement of bone loss was performed using both radiographs, and the mean difference was compared.

The control group consisted of patients who visited the dental clinic for various dental issues and underwent an OPG, which showed the presence of either partial or complete impaction of the lower third molar. However, in this group, the impacted tooth was not extracted. A control panoramic image was performed at least 3–6 months later to assess the oral condition and the evolution of this non-extracted third molar. Since our goal was to examine bone changes in patients with and without extraction, those who did not undergo extraction should have a follow-up panoramic radiograph 3–6 months after their visit. This would help to evaluate their oral status, history, and the condition of the bone distal to the second molar.

### 2.3. Inclusion and Exclusion Criteria

The inclusion criteria for this study were as follows: patients older than 18 years; availability of images depicting the presence of an IMTM; availability of undistorted panoramic images without errors or overlapping; availability of radiographs taken before and after the extraction of the IMTM, with a clearly documented history for the study group; and the presence of a second molar adjacent to the impacted mandibular third molar. The exclusion criteria included images demonstrating an absent or extracted mandibular third molar; patients who had previously undergone chemotherapy, radiotherapy, or bisphosphonate treatment for head and neck tumors; individuals with incomplete records; and those with an incomplete formation of the third molar roots.

### 2.4. Measurement Method

**Figure 1:**
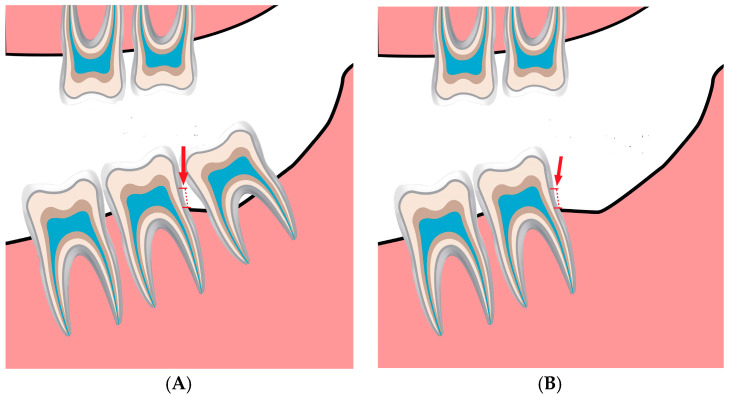
(**A**). The bone level is measured from the cementoenamel junction (red arrow and dotted line) of the second molar downward until the alveolar bone crest before the extraction in the study group. (**B**). The bone level is measured from the cementoenamel junction (red arrow and dotted line) of the second molar downward until the alveolar bone crest after extraction to identify the amount of bone change.

The panoramic images were obtained using a Planmeca ProMax^®^ X-ray unit (Planmeca Oy, 00880 Helsinki, Finland), which was equipped with a digital sensor called Planmeca Dimax 3. The measurement was performed using the Planmeca Romexis^®^ software (Fadente distribution, Badalona, Barcelona, Spain, updated November 2023). Images were taken based on the manufacturer’s recommendations at 64–70 kV and 7–14 mA, depending on the patients’ gender and age. The recommended settings varied for adult females, small adult males, and large adult males. Well-trained doctoral students from the Oral Radiology Department conducted the measurement twice, with a two-month interval between each session. The intra-examiner reliability was calculated using the Kappa statistic [17]. Using the obtained OPGs, the level of the bone was measured from the cementoenamel junction of the second molar to the level of the alveolar crest for the study group before (Figure 1A) extraction and after extraction (Figure 1B).

**Figure 2:**
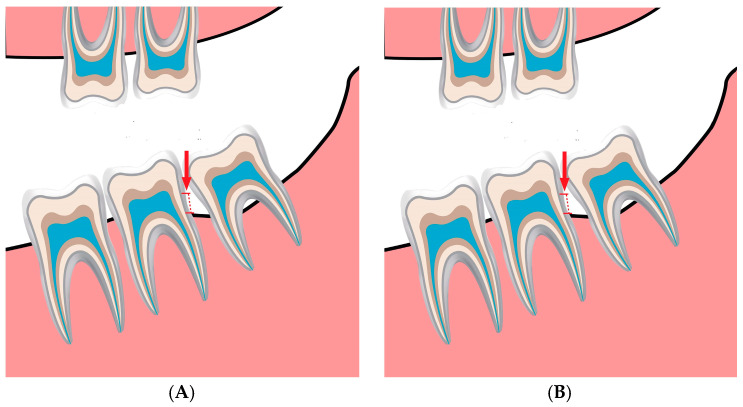
(**A**). The bone level is measured from the cementoenamel junction (red arrow and dotted line) of the second molar downward until the alveolar bone crest in the baseline in the control group. (**B**). The bone level is measured again from the cementoenamel junction (red arrow and dotted line) of the second molar downward until the alveolar bone crest after following up, in which the patients undergo panoramic radiography for other dental tasks, and within a period of 3–6 months to identify the amount of bone change in the control group.

Similarly, bone changes in the control group were measured at baseline (Figure 2A) and after a period of 3–6 months (Figure 2B).

### 2.5. Statistical Analysis

The data were imported into an Excel sheet (Microsoft^®^ Excel^®^ for Microsoft 365 MSO, Version 2307, Microsoft corporation, Washington, DC, USA) for descriptive analysis. All statistical analyses were performed using IBM SPSS Statistics for Windows, version 26 (IBM Corp., Armonk, NY, USA). A chi-squared test was performed to identify differences between the variables. Quantitative variables were expressed as the mean and standard deviation. Since the data were not normally distributed with respect to age and gender, a Wilcoxon test was performed to determine statistical differences in bone-level changes between the two groups. A one-way analysis of variance (ANOVA) was used to investigate differences with respect to gender and age in both groups. According to normality distribution, the U Mann–Whitney test was performed to report statistical significance with respect to the genders among the two groups. The Kruskal–Wallis test was used to report the statistical difference between the groups with respect to the different age groups. A *p*-value of <0.05 was considered statistically significant.

## 3. Results

The mean ages of the patients in the study and control groups were 35.5 ± 15.45 and 33 ± 16.49, respectively. Females and males accounted for 62% and 37.5% of the patients in the study group, respectively, and 55% and 45% of the patients in the control group, respectively. Furthermore, the patients were divided into four subgroups based on age: 18–28, 29–39, 40–50, and ≥51.

### 3.1. Comparison of Bone Levels among the Groups

The bone-level measurements were calculated and reported as the mean and standard deviation for both the study and control groups. The values in the study group before and after extraction were 3.00 ± 1.68 and 2.63 ± 1.75, respectively, indicating a statistically significant difference in favor of bone gain (*p* < 0.0001). In the control group, the value of the baseline radiography was 2.73 ± 1.75, whereas the value of the second radiography was 3.01 ± 1.98, revealing a statistically significant difference in favor of bone loss (*p* < 0.001) (Table 1). The Kappa values were interpreted as follows: low agreement (<0), very slight agreement (0–0.19), slight agreement (0.2–0.39), moderate agreement (04–0.59), substantial agreement (0.6–0.70), and almost perfect agreement (0.8–1) [17] (Table 1).

### 3.2. Comparison of Bone Levels between Genders

Both males and females experienced bone gain in the study group and bone loss in the control group. However, the differences in bone level changes were not statistically significant with respect to gender in the study group (Table 2). Since the data were not normally distributed, the U Mann–Whitney test was performed, and provided no statistical difference between genders of the study group *p*-value 0.747. On the other hand, the control group witnessed bone loss and was higher in males compared to females, with a *p*-value 0.034.

### 3.3. Bone-Level Changes among the Two Groups Based on Age

In the study group, patients between the ages of 29 and 39 years appeared to experience the highest bone gain after extraction compared to those in the other age groups (Table 3). On the other hand, only those aged 40–50 years experienced bone loss after extraction (−1.20 ± 0.14 mm). In the study group, significant differences in bone-level changes were observed among the age groups after extraction (*p* < 0.05; Table 3). Since the data were not normally distributed, a non-parametric Kruskal–Wallis test was performed and resulted in a *p*-value of 0.042, which indicates a statistically significant difference between the 29–39 age group and other groups. Additionally, the post hoc comparison with a Bonferroni test only shows statistical differences between the age groups of 29–39 years old and 40–50 years old. 

In the control group, all age groups presented bone loss at the follow-up evaluation; however, the differences in bone loss among the age groups were not statistically significant (*p*-value 0.794; Table 3). 

### 3.4. Pattern of Impaction

Based on Winter’s classification, the number of IMTMs in this study was 139 out of a total sample of 160. This accounted for 82.28%. Vertical impaction was the most prevalent (41.24%), followed by mesioangular impaction (28.75%) and horizontal impaction (16.62%). No statistically significant difference in the occurrence of different types of impaction was observed in the current study (*p*-value 0.539; Table 4). As the data were not normally distributed, Kruskal–Wallis test is performed and provided a *p*-value 0.794, which indicates no statistical differences between the age groups. Since there is no statistical difference, there was no need to conduct the pos hoc test.

### 3.5. Impaction Side

In both groups, most of the patients demonstrated bilateral impaction, which accounted for 73.8% of the total sample. In the study group, unilateral impaction on the right side was more prevalent than unilateral impaction on the left side (15% and 12.5%, respectively). By contrast, unilateral impaction on the left side was more prevalent than unilateral impaction on the right side in the control group (17.5% and 7.5%, respectively). No statistically significant difference regarding the side of impaction was observed between the two groups (*p*-value = 0.509; Table 5).

## 4. Discussion

Mandibular third molar impaction is one of the most common dental problems encountered in daily dental practice. Surgical removal of an impacted tooth can result in various consequences, which have been reported to occur in 0 to 30% of patients. These complications include pain, trismus, swelling, prolonged bleeding, dry socket, postoperative infection, and paresthesia resulting from an injury to the inferior alveolar nerve [18]. Since pain, swelling, and trismus are among the most common complications of IMTM surgery, Antonelli et al. [19] performed split-mouth randomized clinical trials, in which they investigated the significance of the preoperative prednisone (25 mg/os) administration on such postoperative complications, mainly facial swelling. They compared the use of prednisone in one group with a placebo group at different time intervals and using different methods, including Bollus 3D Face APP for swelling, a visual analog scale for pain, and the calibration of the incisal distance for trismus. According to their outcomes, the preoperative administration of prednisone could improve the overall postoperative complication of third molar surgery, including the facial swelling compared to the placebo group. Their findings are an agreement with those reported by Tiigimae-Saar et al. [20] who stated that the administration of preoperative prednisone contributes to the reduction in edema.

In the present study, the bone level changes in the mandibular second molar (MSM) were compared for patients who underwent extraction of an IMTM and those who did not. The bone level was measured from the cementoenamel junction to the bone crest, as described by Faria et al. [15]. In dental practice, identifying bone loss in the presence of an IMTM is essential for assessing the overall prognosis and planning an appropriate treatment strategy. Despite the possible limitations of OPGs, clinicians continue to rely on them for making interventional decisions [21]. In the current study, a second OPG was available for patients from both groups because they revisited the hospital and OPGs were taken for other dental needs. No significant differences in bone loss were observed among patients in different age groups, although bone loss appeared to be more pronounced in those aged ≥ 51 years in the control group. This finding differed from those reported in the study by Dias et al. [22], wherein statistically significant differences in the severity of bone loss were seen among participants < 50 years old. Interestingly, our findings demonstrated bone loss in the age group of 40–50 years among the patients in the study group. Furthermore, our results agreed with those reported in the study by Fernandes et al. [23], indicating an association between age and a change in the status of the alveolar crest. In the current study, a higher degree of bone loss was observed among males than females, but the difference was not statistically significant. Similar findings were reported in the study by Dias et al. [22], wherein no significant difference in the severity of bone loss distal to the MSM was noted between males and females using panoramic radiography. It should be emphasized that several factors, such as smoking habits, impacted positioning, and a lack of maintenance of proper oral hygiene, can potentially worsen periodontal conditions on the distal aspect of the MSM [24,25]. Therefore, if an impaction is not managed, bone changes in the form of bone loss may occur. Despite variations in IMTM inclination type and other contributing risk factors among the patients in this study, our findings revealed bone loss in patients who did not undergo extraction of the IMTM after an evaluation of their follow-up OPGs. Bone improvement and gain distal to the MSM were observed after the extraction of the IMTM in the study group. These findings aligned with those reported by Passarelli et al. [26], who observed an overall improvement in their patients’ periodontal status following the surgical extraction of an IMTM. The removal of an impacted tooth provides better access for cleaning, thus leading to overall improvement. Similarly, Krausz et al. [21] reported significant improvements in the bone on the distal aspect of the MSM after the extraction of an IMTM. These improvements were clinically and radiographically evaluated using an OPG. Additionally, they noticed mild bone loss in the control group despite variations in the contributing factors, such as the degree of oral hygiene maintenance, which aligned with our findings in the control group. Furthermore, Montero et al. [27] indicated an overall improvement in the periodontal health status adjacent to the MSM after the removal of an IMTM. On the contrary, Kan et al. [28] pointed out the formation of periodontal defects on the distal aspect of the MSM after removing an IMTM. Several studies have identified different patterns of IMTMs in different demographic samples [29,30]. In the present study, the vertical type of impaction was found to be the most prevalent, followed by the mesioangular type. Alsaegh et al. [31] reported a higher prevalence of mesioangular impaction compared to other types in the Arab Emirati population. Similarly, Eshghpour et al. [32] found a higher prevalence of mesioangular impaction in the Iranian population. Prajapati et al. [7] conducted an investigation in the Indian population and reported that mesioangular inclination was more common than the other patterns, including vertical, horizontal, and distoangular inclinations. A greater awareness of the different inclination patterns indicates the need to remove IMTMs and aids in determining the necessary surgical method. The current study revealed a higher number of individuals with bilateral impaction, which was different from the study conducted by Alsaegh et al. [31], wherein a comparable distribution of unilateral and bilateral impactions was reported. However, several studies have reported considerable variations in the occurrence of bilateral and unilateral impaction events among different populations, including Saudi Arabian, Singaporean, Chinese, and Libyan populations [33,34]. Bilateral impaction was found to be predominant in these studies. It should be highlighted that intra-examiner reliability was used instead of inter-examiner reliability when assessing the patients’ OPGs. This decision was made because the assessment was performed concurrently between the examiners, resulting in only one single outcome. Thus, the calculation of the intra-examiner reliability was considered sufficient. To investigate the reproducibility of using OPGs to estimate bone loss on the distal aspect of the MSM, a Kappa test was calculated. Based on the mean of the outcomes, we assigned descriptive categories for the status of bone level changes, including bone loss, bone gain, or no changes in bone level, to report the intra-examiner reliability. These descriptive categories were assigned to translate the quantitative measurements based on panoramic radiography because of the difficulties in reproducing such quantitative measures. Consequently, our Kappa results indicate a substantial agreement of 0.68 between the examiners.

This study has some limitations. The reproducibility of a panoramic radiograph in terms of quantitative measures is questionable due to the inherent limitations of such a method. However, we used this method to assess bone loss distal to the MSM in cases with an IMTM due to its routine use in clinical practice and the expected occurrence of such pathologic conditions. Furthermore, our study solely focused on the radiographic findings without considering clinical parameters, such as those obtained via clinical probing. Thus, further studies correlating findings based on OPGs with three-dimensional imaging, such as cone-beam computed tomography and clinical probing, and the use of a larger sample size are required to validate the findings of the current study. Although a larger sample size could provide more valid findings, our sample size calculation indicated that a total of 24 patients in each group was sufficient for performing the statistical analysis; we increased this number based on the available OPGs that met the inclusion criteria to increase the validity of the evidence. Overall, a larger sample is still advisable to enhance the validity of studies. In addition, the absence of findings from clinical probing is a considerable limitation of our study. 

A strength of this study is that it provides clear evidence about the status of bone level changes in subjects who underwent the extraction of an impacted mandibular third molar compared to those who did not. Hence, the findings could guide clinicians in devising a proper management strategy for this tooth, taking into consideration whether to use OPG alone or combine it with other advanced imaging modalities such as CBCT.

## 5. Conclusions

An increase and improvement in the bone level distal to the mandibular second molar was observed after the extraction of an IMTM when compared to the control group. The findings of this study suggest that, taking into account the routine use, affordability, and convenience of OPG, this method may be beneficial for visualizing a patient’s bone status after the extraction of an impacted molar. 

## Figures and Tables

**Table 1:** Comparison of bone-level changes between the study group and the control group.

Group	Baseline Bone Level Mean SD *	Bone Level after Extraction/Follow-Up Mean SD	*p*-Value
Study group	3.00 ± 1.68	2.63 ± 1.75	0.0001
Control group	2.73 ± 1.75	3.01 ± 1.98	0.001

* SD, standard deviation.

**Table 2:** Bone-level changes in the two groups based on gender.

Group	Gender	Baseline BL ^1^	Follow-Up BL ^2^	Bone Change df (mm) ^3^	*p*-Value
**Study**	Male	3.51 ± 1.89	3.27 ± 2.17	0.24 ± 1.29	0.747
	Female	2.73 ± 1.35	2.29 ± 1.41	0.44 ± 1.13	
**Control**	Male	3.64 ± 1.79	4.12 ± 2.11	−0.48 ± 0.66	0.034
	Female	1.97 ± 1.32	2.11 ± 1.3	−0.13 ± 0.31	

^1^ = bone loss measured before extraction in the study group and at baseline in the control group (non-extraction group); ^2^ = bone loss measured after extraction in the study group and at follow-up in the control group; ^3^ = bone-level difference between the two measurements.

**Table 3:** Bone-level changes in relation to different age groups.

Group	Age Group	Baseline BL	Follow-Up BL	Bone Change df (mm)	*p*-Value
Study	18–28	2.48 ± 1.51	2.18 ± 1.57	0.29 ± 1.07	0.042
	29–39	3.25 ± 1.69	2.29 ± 1.51	0.96 ± 1.05	
	40–50	4.30 ± 3.53	5.50 ± 3.39	−1.20 ±0.14	
	≥51	3.51 ± 1.53	3.45 ± 1.44	0.05 ± 1.34	
Control	18–28	2.93 ± 1.94	3.19 ± 2.13	−0.26 ±0.53	0.794
	29–39	2.28 ± 1.32	2.45 ± 1.50	−0.17 ±0.25	
	40–50	2.20 ± 0.71	2.38 ± 0.46	−0.17 ±0.25	
	≥51	2.58 ± 1.72	3.16 ± 2.35	−0.57 ±0.69	

**Table 4:** Distribution of the impaction pattern according to Winter’s classification.

Classification	Inclination	Study Group		Control Group		Total Sample		*p*-Value
		(N)	(F)	(N)	(F)	(N)	(F)	
Winter’s classification	Vertical	31	44.9%	35	50%	66	41.25%	0.539
	Mesioangular	26	37.7%	20	28.6%	46	28.75%	
	Distoangular	1	1.4%	0	0%	1	1.25%	
	Horizontal	10	14.5%	15	21.4%	25	16.62%	
	Buccally tilted	1	1.4%	0	0%	1	1.25%	
	Total	69	86.25%	70	87.5%	139	82.28%	

**Table 5:** Distribution of impaction type in relation to the side of impaction in the two groups.

Impaction side	**Occurrence**	**Study Group**		**Control Group**		**Total Sample**		***p*-Value**
		**(N)**	**(F)**	**(N)**	**(F)**	**(N)**	**(F)**	
	Right	6	15%	3	7.5%	9	11.3%	0.509
	Left	5	12.5%	7	17.5%	12	15%	
	Bilateral	29	72.5%	30	75%	59	73.8%	

## Data Availability

All relevant data are provided within the article.

## References

[1] Alfadil L., Almajed E. (2020). Prevalence of impacted third molars and the reason for extraction in Saudi Arabia. Saudi Dent. J..

[2] Gupta S., Bhowate R.R., Nigam N., Saxena S. (2011). Evaluation of Impacted Mandibular Third Molars by Panoramic Radiography. ISRN Dent..

[3] Pedro F.L.M., Bandéca M.C., Volpato L.E.R., Marques A.T.C., Borba A.M., de Musis C.R., Borges A.H. (2014). Prevalence of impacted teeth in a Brazilian subpopulation. J. Contemp. Dent. Pract..

[4] Breik O., Grubor D. (2008). The incidence of mandibular third molar impactions in different skeletal face types. Aust. Dent. J..

[5] Padhye M.N., Dabir A.V., Girotra C.S., Pandhi V.H. (2013). Pattern of mandibular third molar impaction in the Indian population: A retrospective clinico-radiographic survey. Oral Surg. Oral Med. Oral Pathol. Oral Radiol..

[6] KalaiSelvan S., Ganesh S.K.N., Natesh P., Moorthy M.S., Niazi T.M., Babu S.S. (2020). Prevalence and Pattern of Impacted Mandibular Third Molar: An Institution-based Retrospective Study. J. Pharm. Bioallied. Sci..

[7] Prajapati V.K., Mitra R., Vinayak K.M. (2017). Pattern of mandibular third molar impaction and its association to caries in mandibular second molar: A clinical variant. Dent. Res. J..

[8] Barone S., Antonelli A., Averta F., Diodati F., Muraca D., Bennardo F., Giudice A. (2021). Does Mandibular Gonial Angle Influence the Eruption Pattern of the Lower Third Molar? A Three-Dimensional Study. J. Clin. Med..

[9] Hassan A. (2010). Pattern of third molar impaction in a Saudi population. Clin. Cosmet. Investig. Dent..

[10] Arora A., Patil B.A., Sodhi A. (2015). Validity of the vertical tube-shift method in determining the relationship between the mandibular third molar roots and the inferior alveolar nerve canal. J. Korean Assoc. Oral Maxillofac. Surg..

[11] Cederhag J., Truedsson A., Alstergren P., Shi X.Q., Hellén-Halme K. (2022). Radiographic imaging in relation to the mandibular third molar: A survey among oral surgeons in Sweden. Clin. Oral Investig..

[12] Shukla S., Chug A., Afrashtehfar K. (2017). Role of cone beam computed tomography in diagnosis and treatment planning in dentistry: An update. J. Int. Soc. Prev. Community Dent..

[13] Ariizumi D., Sakamoto T., Yamamoto M., Nishii Y. (2022). External Root Resorption of Second Molars Due to Impacted Mandibular Third Molars during Orthodontic Retention. Bull. Tokyo Dent. Coll..

[14] Jaju P.P., Jaju S.P. (2015). Cone-beam computed tomography: Time to move from ALARA to ALADA. Imaging Sci. Dent..

[15] Faria A.I., Gallas-Torreira M., López-Ratón M. (2012). Mandibular second molar periodontal healing after impacted third molar extraction in young adults. J. Oral Maxillofac. Surg..

[16] Pham T.A.V., Nguyen N.H. (2019). Periodontal Status of the Adjacent Second Molar after Impacted Mandibular Third Molar Surgical Extraction. Contemp. Clin. Dent..

[17] McHugh M.L. (2012). Interrater reliability: The kappa statistic. Biochem. Med..

[18] Albanese M., Zangani A., Manfrin F., Bertossi D., De Manzoni R., Tomizioli N., Faccioni P., Pardo A. (2023). Influence of Surgical Technique on Post-Operative Complications in the Extraction of the Lower Third Molar: A Retrospective Study. Dent. J..

[19] Antonelli A., Barone S., Bennardo F., Giudice A. (2023). Three-dimensional facial swelling evaluation of pre-operative single-dose of prednisone in third molar surgery: A split-mouth randomized controlled trial. BMC Oral Health.

[20] Tiigimae-Saar J., Leibur E., Tamme T. (2010). The effect of prednisolone on reduction of complaints after impacted third molar removal. Stomatologija.

[21] Krausz A.A., Machtei E.E., Peled M. (2005). Effects of lower third molar extraction on attachment level and alveolar bone height of the adjacent second molar. Int. J. Oral Maxillofac. Surg..

[22] Dias M.J., Franco A., Junqueira J.L., Fayad F.T., Pereira P.H., Oenning A.C. (2020). Marginal bone loss in the second molar related to impacted mandibular third molars: Comparison between panoramic images and cone beam computed tomography. Med. Oral. Patol. Oral Cir. Bucal..

[23] Fernandes I.A., Galvão E.L., Gonçalves P.F., Falci S.G.M. (2022). Impact of the presence of partially erupted third molars on the local radiographic bone condition. Sci. Rep..

[24] Tai S., Zhou Y., Pathak J.L., Piao Z., Zhou L. (2021). The association of mandibular third molar impaction with the dental and periodontal lesions in the adjacent second molars. J. Periodontol..

[25] Tolentino P.H.M.P., Rodrigues L.G., Miranda de Torres É., Franco A., Silva R.F. (2019). Extractions in Patients with Periodontal Diseases and Clinical Decision-Making Process. Acta Stomatol. Croat..

[26] Passarelli P.C., Lajolo C., Pasquantonio G., D’Amato G., Docimo R., Verdugo F., D’Addona A. (2019). Influence of mandibular third molar surgical extraction on the periodontal status of adjacent second molars. J. Periodontol..

[27] Montero J., Mazzaglia G. (2011). Effect of removing an impacted mandibular third molar on the periodontal status of the mandibular second molar. J. Oral Maxillofac. Surg..

[28] Kan K.W., Liu J.K.S., Lo E.C.M., Corbet E.F., Leung W.K. (2002). Residual periodontal defects distal to the mandibular second molar 6–36 months after impacted third molar extraction. J. Clin. Periodontol..

[29] Al-Dajani M., Abouonq A.O., Almohammadi T.A., Alruwaili M.K., Alswilem R.O., Alzoubi I.A. (2017). A Cohort Study of the Patterns of Third Molar Impaction in Panoramic Radiographs in Saudi Population. Open Dent. J..

[30] Yilmaz S., Adisen M.Z., Misirlioglu M., Yorubulut S. (2016). Assessment of Third Molar Impaction Pattern and Associated Clinical Symptoms in a Central Anatolian Turkish Population. Med. Princ. Pract..

[31] Alsaegh M.A., Abushweme D.A., Ahmed K.O., Ahmed S.O. (2022). The pattern of mandibular third molar impaction and its relationship with the development of distal caries in adjacent second molars among Emiratis: A retrospective study. BMC Oral Health.

[32] Eshghpour M., Nezadi A., Moradi A., Shamsabadi R.M., Rezaei N.M., Nejat A. (2014). Pattern of mandibular third molar impaction: A cross-sectional study in northeast of Iran. Niger J. Clin. Pract..

[33] Zaman M.U., Almutairi N.S., Abdulrahman Alnashwan M., Albogami S.M., Alkhammash N.M., Alam M.K. (2021). Pattern of Mandibular Third Molar Impaction in Nonsyndromic 17760 Patients: A Retrospective Study among Saudi Population in Central Region, Saudi Arabia. Biomed. Res. Int..

[34] Quek S.L., Tay C.K., Tay K.H., Toh S.L., Lim K.C. (2003). Pattern of third molar impaction in a Singapore Chinese population: A retrospective radiographic survey. Int. J. Oral Maxillofac. Surg..

